# Histone Deacetylase Inhibitor Impairs Plasminogen Activator Inhibitor-1 Expression via Inhibiting TNF-****α****-Activated MAPK/AP-1 Signaling Cascade

**DOI:** 10.1155/2014/231012

**Published:** 2014-02-23

**Authors:** Wei-Lin Chen, Joen-Rong Sheu, Che-Jen Hsiao, Shih-Hsin Hsiao, Chi-Li Chung, George Hsiao

**Affiliations:** ^1^Graduate Institute of Medical Sciences and Department of Pharmacology, College of Medicine, Taipei Medical University, 250 Wu-Hsing Street, Taipei 110-31, Taiwan; ^2^School of Respiratory Therapy, College of Medicine, Taipei Medical University, Taipei 110-31, Taiwan; ^3^Division of Pulmonary Medicine, Department of Internal Medicine, Taipei Medical University Hospital, Taipei 110-31, Taiwan

## Abstract

Tumor necrosis factor-(TNF-)-*α* upregulates plasminogen activator inhibitor-(PAI-) 1 expression in pleural mesothelial cells (PMCs), contributing to fibrin deposition and pleural fibrosis. Histone deacetylases (HDACs) have been found implicated in fibrogenesis. However, the roles of TNF-*α* or HDAC in the regulation of PAI-1 expression have not been well investigated. We aimed to examine the effects and mechanisms of HDAC inhibition on TNF-*α*-induced PAI-1 expression in human PMCs. MeT-5A human PMCs were treated with TNF-*α* in the presence or absence of the *m*-carboxycinnamic acid bishydroxamide (CBHA), an HDAC class II inhibitor, and the HDAC activity, PAI-1 protein expression, mRNA, and activated signalings were analyzed. CBHA abrogated TNF-*α*-induced HDAC activity, PAI-1 protein and, mRNA expression in MeT-5A cells. Moreover, CBHA significantly enhanced mitogen-activated protein kinase phosphatase-(MKP-) 5/MKP-1 expression and inhibited p38/JNK activations, ATF2/c-Jun translocation, and PAI-1 promoter activity. Altogether, our data suggest that HDAC inhibition may abrogate TNF-*α*-activated MAPK/AP-1 signaling and PAI-1 expression in human PMCs. Given the antifibrotic effect through PAI-1 abrogation, CBHA may be utilized as a novel agent in the treatment of fibrotic diseases.

## 1. Introduction

Pleural fibrosis is a common sequel in a variety of inflammatory pleural effusions, such as empyema and tuberculous pleurisy [1]. In general, fibrin turnover in the pleural space is greatly affected by equilibrium between plasminogen activators (PAs) and plasminogen activator inhibitors (PAIs) [2]. The elevation of PAI-1 level in the pleural fluid decreases the fibrinolytic activity in the pleural space and leads to fibrin deposition and subsequent pleural fibrosis [3]. Tumor necrosis factor (TNF)-*α*, a potent inflammatory mediator involved in the pathogenesis of infectious pleural effusion [4], has been proved to stimulate the production of PAI-1 in human pleural mesothelial cells (PMCs) [5]. Previous studies demonstrated a positive correlation between the values of TNF-*α* and PAI-1 in tuberculous pleural effusion, and both proteins are significantly higher in the pleural fluids of those with residual pleural fibrosis [6, 7]. These findings suggest that TNF-*α* and PAI-1 are implicated in fibrogenesis of the pleural space. However, the underlying mechanism of TNF-*α*-induced PAI-1 expression in the pleural space is not clearly understood.

Histone acetyltransferase (HAT) and histone deacetylase (HDAC) regulate the acetylation of both histone and nonhistone proteins and play critical roles in the modulation of gene expression in multiple cellular processes from signaling, transcription, and mRNA stability to protein degradation [8, 9]. Altered activities of HAT and HDAC in a given cell may cause aberrant gene expression and lead to various pathological processes, such as cancer [10], inflammation [11], and fibrosis [12]. Recently, HDAC inhibitors have been found to correct aberrant protein acetylation and gene expression and are considered as promising therapeutic agents for malignancy [13] and cardiac fibrosis [14].

Moreover, our recent study demonstrated that m-carboxycinnamic acid bishydroxamide (CBHA), a hybrid-polar HDAC inhibitor, attenuates transforming growth factor (TGF)-*β*1-induced PAI-1 expression in human PMCs [15]. In addition, another HDAC inhibitor Trichostatin A (TSA) has been proved to block TGF-*β*1-induced fibroblast-myofibroblast differentiation through inhibition of phosphorylation of Akt and subsequent expression of *α*-SMA [16]. However, to our knowledge, the role of the proinflammatory cytokine, such as TNF-*α*, or HDAC in the regulation of PAI-1 expression in PMCs and the effect of HDAC inhibition on pleural fibrogenesis have not been well investigated. Therefore, in the present study, we used CBHA, a class II HDAC inhibitor, to explore the effects of HDAC inhibition on TNF-*α*-induced PAI-1 expression in human PMCs.

## 2. Materials and Methods

### 2.1. Reagents

Recombinant human TNF-*α* was purchased from Pepro Tech EC (London, UK). The PAI-1 reporter plasmid (p800Luc) that contains 800 bp of the proximal promoter sequences of human PAI-1 gene was a generous gift of Professor Daniel Rifkin (New York University) [15]. Except for PAI-1 (BD Biosciences, San Jose, CA), other antibodies were purchased from Cell Signaling Technology (Beverly, MA). Doxycycline and all of the other chemical reagents were purchased from Sigma-Aldrich (St. Louis, MO). CBHA, SB203580, SP600125, PD98059, LY294002, and parthenolide were obtained from Calbiochem (San Diego, CA).

### 2.2. Cell Line and Primary Culture of Human Pleural Mesothelial Cells

The Met-5A human pleural mesothelial cell line was obtained from American Type Culture Collection (ATCC, Manassas, VA). Cells culture was performed as described in our previous report [17]. Primary cultured human PMCs were harvested from the pleural fluids of patients with congestive heart failure. Ethics approval was obtained from the Institutional Review Board (IRB number: CRC-05-11-01) of Taipei Medical University, and the written informed consent was acquired. The human pleural fluids were centrifuged and cells were grown in medium 199 containing 10% FBS at 37°C in the humidified incubator of 5% CO_2_. Mesothelial cells were used at passages three to six and were characterized by the cobblestone morphology, the presence of cytokeratin, and the absence of factor VIII [18].

### 2.3. Total Cellular HDAC Enzyme Activity Assay

Total HDAC enzyme activity was determined by using the HDAC fluorometric cellular activity assay (Enzo Life Sciences) according to the manufacturer's protocol. MeT-5A cells were treated with TNF-*α* for the indicated times or pretreated with CBHA for 15 min before stimulation with TNF-*α* for 2 h. The fluorescence intensity was measured on a fluorometric reader using excitation/emission wavelength of 360/460 nm. The results of cellular HDAC activity were presented as relative multiples as compared to the control.

### 2.4. Differential Protein Fractionation and Western Blot Analysis

The cellular lysates were performed as previously mentioned [17], and nuclear extracts were prepared using the NE-PER kit (Pierce, Rockford, IL). The proteins were separated in denaturing sodium dodecyl sulfate (SDS) polyacrylamide gels and electrophoretically transferred onto nitrocellulose membranes. Blotting membranes were incubated with specific primary and HRP-conjugated secondary antibodies. The quantitative densitometric analysis was performed as previously described [17].

### 2.5. Reverse Transcription-Polymerase Chain Reaction (RT-PCR)

Total RNA was isolated from Met-5A cells using the TRIzol reagent (GIBCO) and RNA (1 *μ*g) was used for cDNA synthesis (Super Script On-Step RT-PCR system, GIBCOTM). The cDNAs were amplified using the specific primers and the quantitative analyses were performed as previously described [17].

### 2.6. Transfection and Luciferase Activity Assay

MeT-5A cells were transfected with the PAI-1 reporter plasmid using the Lipofectamine 2000 transfection reagent (Invitrogen, Carlsbad, CA). After 24 h incubation with the transfection reagent in serum and antibiotic free medium, cells were treated with CBHA for 15 min and subsequently stimulated with TNF-*α* for another 24 h. PAI-1 luciferase activity was measured as described previously [15].

### 2.7. Statistical Analyses

Data analyses were performed with SigmaStat 3.5 (SYSTAT Software, San Jose, CA). Quantitative data are presented as means ± SEM. The statistical analysis was performed using one-way ANOVA. The Student-Newman-Keuls test was used if group comparisons showed a significance difference. *P* < 0.05 was considered statistically significant.

## 3. Results

### 3.1. Effect of CBHA on TNF-*α*-Induced HDAC Activity and PAI-1 Expression

**Figure 1:**
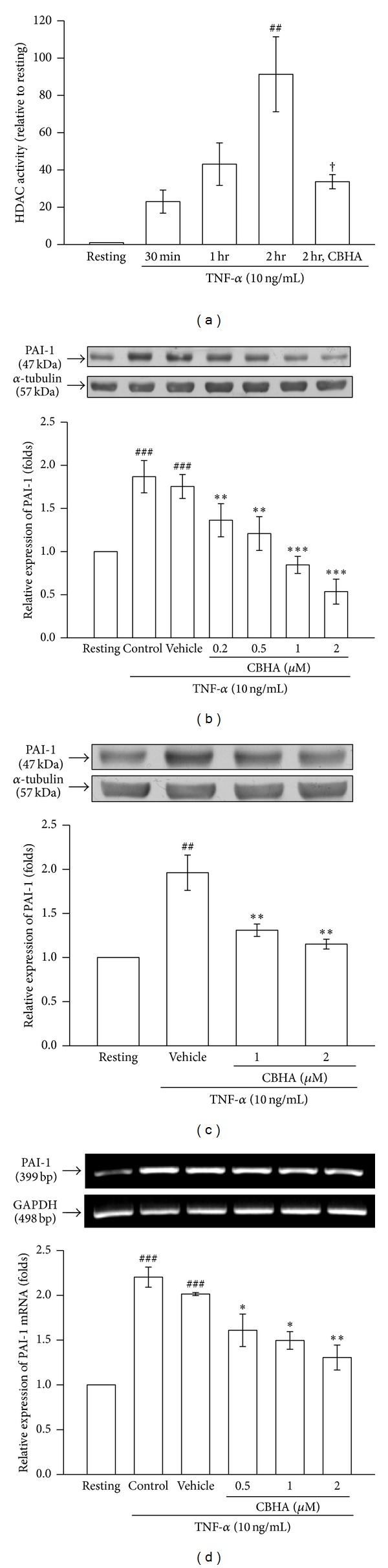
Effect of CBHA on HDAC activity and PAI-1 expression in human pleural mesothelial cells. (a) MeT-5A cells were treated with TNF-*α* (10 ng/mL) for the indicated times or pretreated with CBHA (1 *μ*M) for 15 min before stimulation with TNF-*α* for 2 hours. Total HDAC enzyme activity was determined by using the HDAC fluorometric cellular activity assay. (b) MeT-5A cells and (c) primary cultured human pleural mesothelial cells were pretreated with CBHA, respectively, followed by stimulation with TNF-*α* (10 ng/mL) for 24 h. The levels of PAI-1 were assessed by Western blot. (d) MeT-5A cells were pretreated with CBHA (0.5–2 *μ*M), followed by stimulation with TNF-*α* (10 ng/mL) for 6 h. PAI-1 mRNA concentrations were analyzed by semiquantitative reverse transcriptase PCR and normalized with glyceraldehyde 3-phosphate dehydrogenase (GAPDH) mRNA. Band intensity was quantified as described in Section 2. Data are shown as mean ± SEM of three independent experiments. ^##^ *P* < 0.01 and ^###^ *P* < 0.001 compared with the resting group; *P < 0.05, **P < 0.01, and ***P < 0.001 compared with the vehicle (DMSO) group; ^†^ *P* < 0.05 compared with the 2 hr TNF-*α*-treated group.

To determine the functional relevance of CBHA, we assessed whether CBHA was able to inhibit the pan-HDAC activity in MeT-5A cells. As shown in Figure 1(a), TNF-*α* (10 ng/mL) stimulated significant increase by up to approximately 91 folds in cellular HDAC activity in MeT-5A cells at 2 h, compared with the resting condition. Pretreatment with CBHA (1 *μ*M) strongly inhibited TNF-*α*-induced HDAC activity.

As shown in Figure 1(b), CBHA concentration (0.2, 0.5, 1, and 2 *μ*M) dependently inhibited the TNF-*α*-stimulated production of PAI-1. Consistently, this inhibitory capacity of CBHA (1 and 2 *μ*M) on PAI-1 expression was also verified in the purified human primary cultured PMCs (Figure 1(c)). 

Moreover, TNF-*α* significantly increased expression of PAI-1 mRNA in MeT-5A cells as compared with the resting condition. Pretreatment with CBHA (0.5, 1, and 2 *μ*M) significantly decreased TNF-*α*-induced PAI-1 mRNA expression (Figure 1(d)). These results indicated that CBHA markedly suppresses TNF-*α*-induced PAI-1 protein synthesis through inhibition of PAI-1 gene expression in MeT-5A cells.

### 3.2. Effect of Signaling Inhibitors and CBHA on TNF-*α*-Induced Activation and PAI-1 Expression

**Figure 2:**
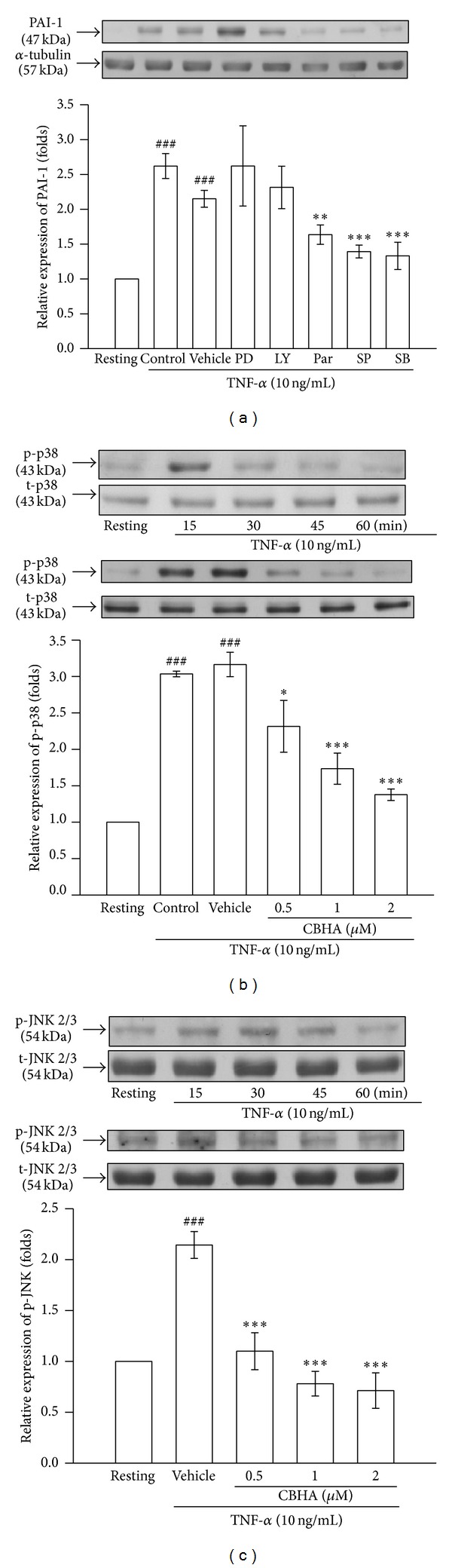
Effect of CBHA on TNF-*α*-activated signalings in MeT-5A cells. (a) Cells were pretreated with vehicle, PD98059 (PD, 20 *μ*M), LY294002 (LY, 10 *μ*M), Parthenolide (Par, 10 *μ*M), SP600125 (SP, 10 *μ*M), and SB203580 (SB, 20 *μ*M), and then stimulated with TNF-*α* (10 ng/mL) for 24 h. PAI-1 protein expression was assessed by Western blot. (b) and (c) Cells were treated with TNF-*α* for the indicated times (upper panel) or pretreated with vehicle or CBHA (0.5–2 *μ*M) for 15 min followed by TNF-*α* administration (lower panel). Phosphorylation of (b) p38 and (c) JNK MAPKs was analyzed by Western blotting with antibodies specific for either phosphorylated or total proteins.

In order to verify the inhibitory mechanism of CBHA on TNF-*α*-induced PAI-1 expression in MeT-5A cells, we examined several TNF-*α*-dependent and -independent signaling pathways, including NF-*κ*B, PI3K/AKT, or MAPKs, by using their specific pharmacologic inhibitors. As shown in Figure 2(a), PAI-1 expression induced by TNF-*α* was markedly attenuated by pretreatment with an IKK inhibitor (parthenolide), a p38 MAPK inhibitor (SB203580), and a JNK inhibitor (SP600125). Neither a MEK inhibitor (PD98059) nor a PI3K inhibitor (LY294002) affected TNF-*α*-stimulated PAI-1 protein production. Consistently, TNF-*α* significantly induced phosphorylation of both p38 and JNK (2/3) MAPK within 15 min and 30 min, compared with the resting condition (Figures 2(b) and 2(c), upper panel), respectively. Moreover, the elevation of p38 and JNK phosphorylation was strongly attenuated by CBHA in a concentration-dependent manner (Figures 2(b) and 2(c), lower panel). However, pretreatment with different concentrations of CBHA had no significant effect on TNF-*α*-activated I*κ*B*α* phosphorylation and degradation (data not shown). These results suggested that CBHA suppressed TNF-*α*-induced PAI-1 expression via inhibiting p38/JNK phosphorylation, but not I*κ*B*α* degredation in MeT-5A cells.

### 3.3. Effect of CBHA on TNF-*α*-Induced MKP Expression

**Figure 3:**
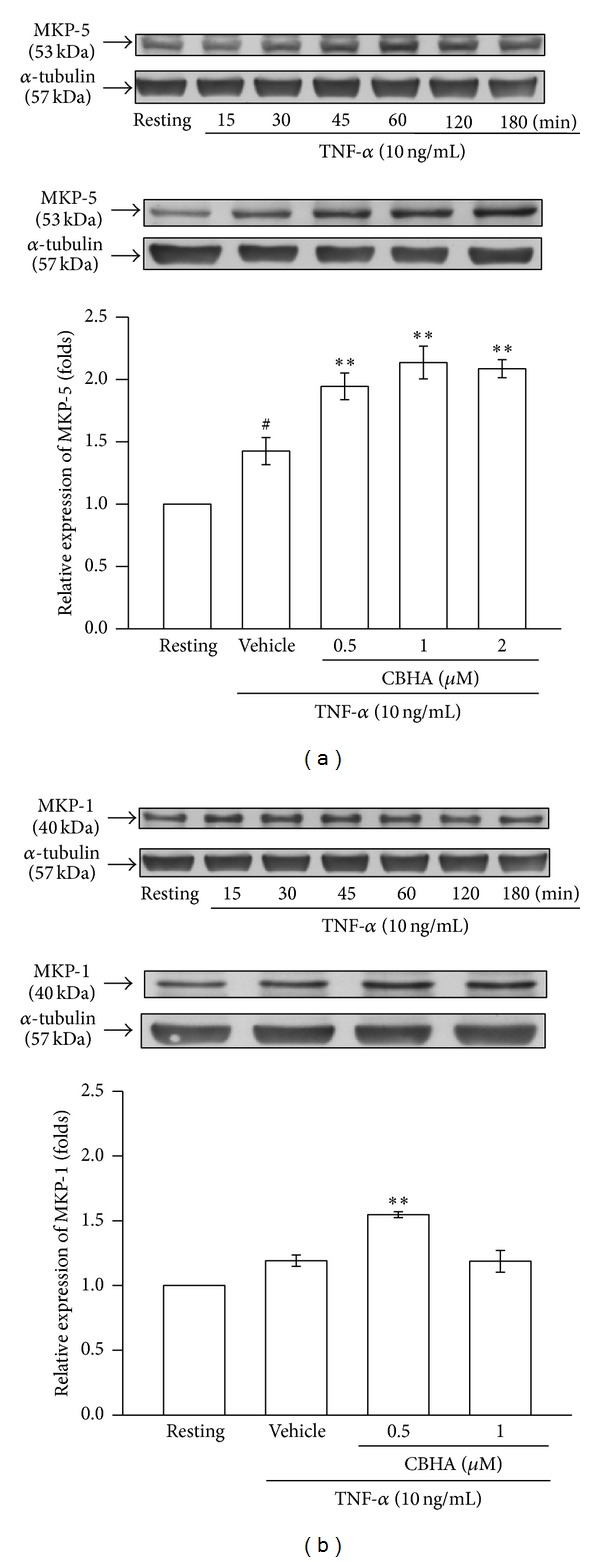
Effect of CBHA on TNF-*α*-induced MKP expression in MeT-5A cells. (a) and (b) Cells were treated with TNF-*α* for the indicated times (upper panel) or pretreated with CBHA (0.5–2 *μ*M) for 15 min followed by TNF-*α* stimulation for 60 min and 15 min (lower panel), respectively. The expression of MKP-5/MKP-1 was analyzed by Western blotting. Relative multiples of densitometric data are expressed as mean ± SEM of three independent experiments. ^#^ *P* < 0.05 compared with the resting group; **P < 0.01 and compared with vehicle group.

MAPK phosphatase (MPK) is known to dephosphorylate and deactivate various members of the MAPK family, including p38 and JNK MAPK. To clarify the inhibitory mechanism of CBHA on activation of p38/JNK MAPK, we further examined the effect of CBHA on TNF-*α*-induced MKP-5 and MKP-1 expression. As shown in the time course studies in Figures 3(a) and 3(b) (upper panels), TNF-*α* increased MKP-5 and MKP-1 expression within 60 min, compared to the resting in MeT-5A cells. The inducing effect was significantly enhanced by pretreatment with CBHA (Figures 3(a) and 3(b), lower panel), especially MKP-5 enhancement, indicating that CBHA inhibits TNF-*α*-activated p38/JNK phosphorylation through increasing MKP-5/MKP-1 expression.

### 3.4. Effect of CBHA on TNF-*α*-Induced Activator Protein (AP)-1 Activation and PAI-1 Promoter Activity

**Figure 4:**
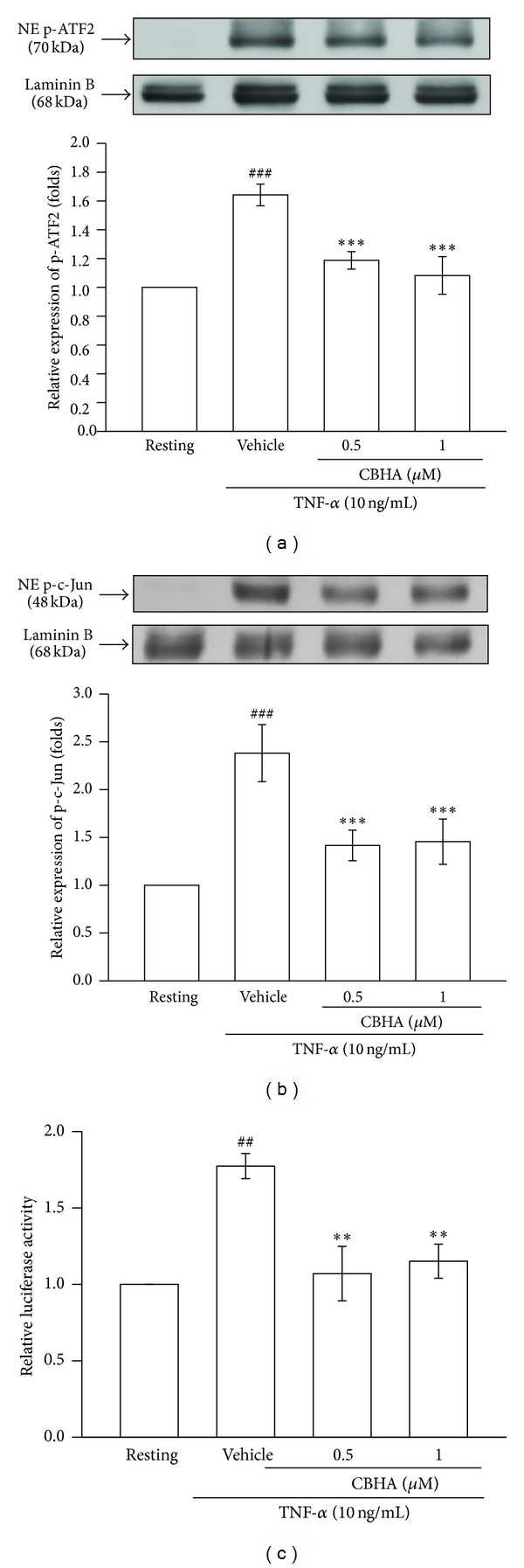
Effect of CBHA on TNF-*α*-induced activator protein (AP)-1 activity and PAI-1 promoter in MeT-5A cells. (a) and (b) Cells were pretreated with vehicle or CBHA (0.5–1 *μ*M) for 15 min, followed by treatment with TNF-*α* (10 ng/mL) for 60 min. The nuclear amount of phosphorylated (a) ATF-2 and (b) c-Jun was detected by Western blot analysis of nuclear extracts (NE) with specific antibodies. (c) Cells were transfected with the specific PAI-1 reporter plasmid (p800Luc), together with internal plasmid (Renilla). Transfected cells were pretreated with vehicle or CBHA (0.5–2 *μ*M) for 15 min and then stimulated with TNF-*α* (10 ng/mL) for 24 h. The luciferase activity of PAI-1 reporter gene was assessed. Data are representative of three to four experiments. ^##^ *P* < 0.01, ^###^ *P* < 0.001 compared with the resting group; **P < 0.01, ***P < 0.001 compared with the vehicle group.

We proposed that CBHA may consistently affect PAI-1 gene transcription via disruption of the activation and nuclear translocation of the downstream transcription factor AP-1. Therefore, the activation of AP-1 constituents including ATF2 and c-Jun in nuclear extracts was analyzed by Western blotting. As shown in Figures 4(a) and 4(b), TNF-*α* increased nuclear activation of ATF2 and c-Jun as compared with the resting condition. Pretreatment with CBHA attenuated TNF-*α*-induced phosphorylation of both ATF2 and c-Jun in nuclear extracts. These results indicated that CBHA may inhibit TNF-*α*-induced ATF2 and c-Jun nuclear activation and thereby impair AP-1 transcriptional activity and PAI-1 gene expression.

Next, to evaluate whether the inhibition of PAI-1 gene expression by CBHA occurred at the transcriptional level, we studied the effect of CBHA on TNF-*α*-induced PAI-1 promoter activity using p800luc reporter plasmid [19]. TNF-*α* significantly increased the luciferase activity compared with the resting condition. Pretreatment of cells with various concentrations of CBHA caused a significant inhibition of TNF-*α*-induced PAI-1 promoter activity (Figure 4(c)).

## 4. Discussion

In the present study, we investigated the potential mechanisms underlying the antifibrotic activity of HDAC inhibitor in human pleural mesothelial cells. Our study demonstrated that HDAC inhibition with CBHA may downregulate TNF-*α*-induced PAI-1 expression in human PMCs. CBHA may suppress TNF-*α*-stimulated cellular HDAC activity, increase MKP-5/MKP-1 expression, and thereby repress p38/JNK and ATF2/c-Jun activation and decrease PAI-1 promoter activity and gene expression. To our knowledge, this is the first study to show that TNF-*α* increases cellular HDAC activity and that HDAC inhibition abrogates TNF-*α*-activated cellular signalings, PAI-1 expression.

HDAC activation is an important mechanism in modulation of gene expression [20]. However, it has not been shown whether HDAC plays a role in TNF-*α*-induced PAI-1 expression in human PMCs. A previous report showed that pan-HDAC inhibitors amplified PAI-1 expression in LPS-stimulated mouse macrophages [21]. In contrast, our recent study revealed that the HDAC inhibitor CBHA attenuated TGF-*β*1-induced PAI-1 expression in human PMCs [15], suggesting that the role of HDAC in regulation of PAI-1 expression may be stimulant- or cell type-specific. On the other hand, in line with previous reports [2, 4], the present study demonstrated that TNF-*α* increased PAI-1 expression in human PMCs and that this upregulation effect was markedly attenuated by CBHA.

The molecular mechanisms by which signaling pathways are involved in the regulation of PAI-1 expression remain to be determined in TNF-*α*-stimulated human PMCs. TNF-*α* elicits inflammatory response via several signal transduction pathways, including NF-*κ*B and MAPK pathways [22], and it is well known that the PAI-1 promoter contains binding sites for Smads, NF-*κ*B, and AP-1 [19]. HDAC inhibitors have been shown to prevent renal interstitial fibrosis through inhibition of NF-*κ*B activity [23]. Also, a previous study reported that HDAC4 silencing blocked TGF-*β*1-induced *α*-SMA expression in fibroblasts through impairing AKT phosphorylation [16]. Alternatively, our study showed that inhibition of HDAC activity with CBHA ablated TNF-*α*-stimulated phosphorylation of p38 and JNK MAPK, but not NF-*κ*B activation (data not shown).

**Figure 5:**
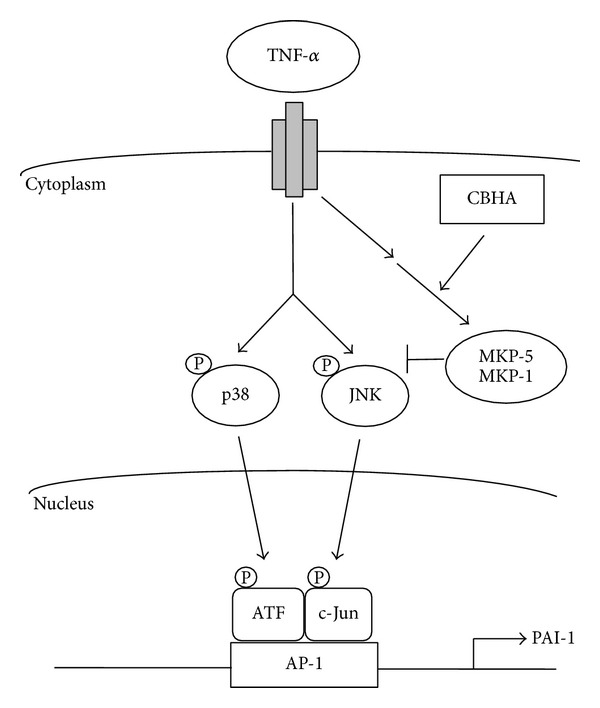
Schematic diagram shows that CBHA abrogates TNF-*α*-induced PAI-1 expression in human pleural mesothelial cells through enhancement of MKP-5/MKP-1 expression and repression of MAPK/AP-1 signal pathway (see test for further explanation). Single arrow, established signal pathway; double arrow, possible signal pathway. (P), phosphorylated.

Additionally, the activity of the MAPK family is determined by a dynamic balance between phosphorylation and dephosphorylation. Among the numerous MAPK phosphatases, MKP-5 and MKP-1 are known to dephosphorylate and inactivate p38 and JNK MAPK [24]. Our data demonstrated that CBHA significantly increased MKP-5 and MKP-1 expression induced by TNF-*α*, suggesting that CBHA may inactivate MAPK signaling via enhancing MKP-5 and MKP-1 expression in human PMCs. Furthermore, as a transcription factor of MAPK signaling, AP-1 is composed of either homodimers or heterodimers between Fos, Jun, and ATF2 family members, which are activated by phosphorylated MAPKs and dimerize with each other to bind to AP-1 promoter site [25]. Correspondingly, the present study demonstrated that CBHA markedly repressed TNF-*α*-induced AP-1 transcriptional activity via disruption of ATF2/c-Jun trans-activation into the nucleus and PAI-1 promoter activity. All these findings suggested that CBHA may abrogate TNF-*α*-induced PAI-1 expression in MeT-5A cells through induction of MKP-5/MKP-1 expression and repression of MAPK/AP-1 signal pathway (Figure 5). However, this study could not exclude other mechanisms such as epigenetic histone acetylation modulation and transcriptional and posttranscriptional regulation [15, 26], in the control of PAI-1 expression.

Collectively, CBHA attenuated PAI-1 expression via inhibition of TNF-*α*-activated signaling, indicating a potential antifibrotic effect of HDAC inhibitors. A recently published study demonstrated that MPT0E014, a novel HDAC inhibitor, decreased the expression of angiotensin II receptor and TGF-*β*1 in cardiac fibroblasts, inhibited their proliferation and migration, and reduced cardiac fibrosis in heart failure rats, which highly signified the direct antifibrotic activity through HDAC inhibition [14]. In parallel, our study disclosed the downregulation effect of CBHA on the profibrotic mediator PAI-1 in pleural mesothelial cells and verified the indirect action of HDAC inhibitors on pleural fibrosis. However, in contrast to the previous study [14], the current work did not explore the functional regulation of fibrogenesis by HDAC inhibition but focused on the modulation of TNF-*α*-mediated signaling by CBHA at multiple levels, which may revalidate the pluripotent effects of HDAC inhibitors [9]. Further *in vivo* studies are needed to examine the direct antifibrotic effect of CBHA.

In conclusion, the present study demonstrated that inhibition of HDAC activity with CBHA may increase MKP-5/MKP-1 expression, abrogate TNF-*α*-activated MAPK/AP-1 signaling and thereby impair PAI-1 expression in human PMCs. Given the antifibrotic effect through PAI-1 abrogation, CBHA may be utilized as a novel agent in the treatment of fibrotic diseases.
